# Effects of cosmetics on the skin microbiome of facial cheeks with different hydration levels

**DOI:** 10.1002/mbo3.557

**Published:** 2017-11-29

**Authors:** Hyo Jung Lee, Sang Eun Jeong, Soyoun Lee, Sungwoo Kim, Hyuntak Han, Che Ok Jeon

**Affiliations:** ^1^ Department of Biology Kunsan National University Gunsan Korea; ^2^ Department of Life Science Chung‐Ang University Seoul Korea; ^3^ Coway Cosmetics R&D Center Seoul Korea

**Keywords:** cosmetics, facial skin microbiome, LEfSe, *Propionibacterium*, *Ralstonia*, skin hydration

## Abstract

Basic cosmetics was used by volunteers belonging to high (HHG) and low (LHG) hydration groups for 4 weeks, and bacterial communities and biophysical parameters in facial skin were analyzed. Hydration level increases and transepidermal water loss and roughness decreases were observed in both groups after cosmetic use. Bacterial diversity was greater in LHG than HHG, and increased after cosmetic use in both groups. Bray–Curtis dissimilarities that were higher in LHG than HHG increased in HHG after cosmetic use, whereas they decreased in LHG. The phyla *Actinobacteria*,* Proteobacteria*,* Firmicutes*, and *Bacteroidetes* and the genera *Propionibacterium*,* Ralstonia*,* Burkholderia*,* Staphylococcus*,* Corynebacterium*,* Cupriavidus*, and *Pelomonas* were identified as common groups and they were not significantly different between LHG and HHG except for *Propionibacterium* that was more abundant in HHG. After cosmetic use, *Propionibacterium*,* Staphylococcus*, and *Corynebacterium* decreased, whereas *Ralstonia*, not a core genus, increased, as did KEGG categories of lipid metabolism and xenobiotics biodegradation and metabolism, suggesting that *Ralstonia* in skin may have the ability to metabolize cosmetics components. Bacterial communities after cosmetic use were different from those in both LHG and HHG before the cosmetic use, indicating that bacterial communities in LHG were not shifted to resemble those in HHG by cosmetics use.

## 1 INTRODUCTION

Human skin is the front line of defenses against external infectious or toxic substances, and is an environmental habitat that various microorganisms, including bacteria, fungi, yeasts, and viruses, can colonize (Roth & James, 1988; Schommer & Gallo, 2013). Human skin is a complex ecosystem with various microenvironmental conditions, and thus, skin microbial communities are very diverse and complex (Oh et al., 2014; Schommer & Gallo, 2013). Skin structures such as hair follicles, sebaceous glands, eccrine and apocrine sweat glands as well as subepidermal skin compartments, provide distinct biological niches that are colonized by their own unique skin microbiota (Costello et al., 2009; Grice et al., 2008; Nakatsuji et al., 2013; Oh et al., 2014). The current understanding is that most of these skin microbes are harmless or commensal organisms that play essential roles in inhibiting colonization by pathogenic microbes or modulating innate and adaptive immune systems (Belkaid & Segre, 2014; Grice, 2015; Rosenthal, Goldberg, Aiello, Larson, & Foxman, 2011; Scharschmidt & Fischbach, 2013).

Bacterial community analyses using phylogenetic marker genes and shot gun metagenomic surveys have revealed that the physical and chemical characteristics of the skin sites, such as pH, temperature, moisture content, sebum content, and topography have an influence on microbial communities and abundance (Findley et al., 2013; Gri Grice & Segre, 2011; Grice et al., 2009; Kong, 2011; Perez et al., 2016). Besides them, various factors, including the use of antibiotics, cosmetics, soaps, personal care products, and living conditions such as life styles and alimentations can have influence on the skin microbiome (Perez et al., 2016). Skin microbial communities have been reported to be site‐, individual‐, and race‐specific and are largely stable over time, despite the human skin's exposure to different external environments such as climates (Leung, Wilkins, & Lee, 2015; Oh, Byrd, Park, Kong, & Segre, 2016; Oh et al., 2014). It was also reported that the skin microbiome was clearly different depending on the ethnic races, which may be because endogenous (immune status, genetic characters, and skin properties) and exogenous (foods and life styles) factors are different depending on ethnicity (Alexis & Alam, 2012; Pappas, Fantasia, & Chen, 2013; Perez et al., 2016). Many skin microbiome studies such as in patients with primary immunodeficiencies (Oh et al., 2013), normal and sensitive skin (Hillion et al., 2013), male and female individuals (Fierer, Hamady, Lauber, & Knight, 2008; Ying et al., 2015), patients with atopic dermatitis (Sator, Schmidt, & Hönigsmann, 2003), and twins (Si, Lee, Park, Sung, & Ko, 2015) have been performed, and their results have suggested that the delicate balance of the skin microbiome may have a strong influence on the functional differences between healthy skin and diseased or damaged skin (Kong, 2011; Rosenthal et al., 2011; Zeeuwen, Kleerebezem, Timmerman, & Schalkwijk, 2013).

The hydration level in the surface layer of the human skin, the stratum corneum (SC), is an important factor affecting the biophysical properties and function of the skin barrier (Wertz, 1996). Dry skin with low hydration level is generally accepted to be prone to having wrinkled, scaly, or rough properties, with the possible presence of cracking, reddening or itching, and less flexibility compared to normal skin (Flynn, Petros, Clark, & Viehman, 2001). Dry skin is susceptible to skin aging or damage, and aged skin does not easily recover without treatment (Rawlings & Matts, 2005). In particular, the human face can remain in a dry state during the whole lifespan because the face is directly exposed to the external environment and some measures to maintain an appropriate hydration level of facial skin may be necessary for the prevention of skin problems or aging in some people. The use of basic skin care products (called basic cosmetics in this study) can be a way to maintain an appropriate facial skin hydration level because lipids or oils, major components in basic cosmetics, form an occlusive layer on skin (Proksch & Lachapelle, 2005; Sator et al., 2003) or many other small molecules in basic cosmetics alleviate dry skin symptoms (Björklund, Engblom, Thuresson, & Sparr, 2013; Nowacka, Douezan, Wadsö, Topgaard, & Sparr, 2012). Hydration has a substantial effect on skin biophysical parameters such as skin transepidermal water loss (TEWL) and roughness, as well as the skin microbial community. However, the relationship between hydration level and the microbial community in the human skin has not been extensively studied, and studies have been only shown that the normal resident skin microbiome varies significantly between human body sites with different hydration levels (Grice et al., 2009; Perez et al., 2016). Moreover, there are no studies investigating the effects of basic cosmetics that increase skin hydration level on the skin microbiome. As basic cosmetics increase the skin hydration level of dry skin, the microbial communities in dry skin may shift to resemble those in normal skin after using basic cosmetics. Therefore, in this study we compared bacterial communities of facial skin with two different skin hydration levels (high hydration group, HHG; low hydration group, LHG), together with measurements of skin biophysical parameters (skin hydration, TEWL, and roughness). In addition, we investigated the effects of basic cosmetics on skin biophysical parameters and the facial skin microbiome in the two groups.

## 2 MATERIALS AND METHODS

### 2.1 Subjects and study design

Ethics approval for this study was obtained from the Dermapro Ltd. Institutional Review Board (reference number 1‐220777‐A‐N‐02‐DICN15101), and each volunteer gave written informed consent before participating. All protocols and procedures used in this study were conducted according to the Declaration of Helsinki. Participants with the following characteristics were excluded from this study: (1) were pregnant or lactating; (2) had performed a similar study within 3 months; (3) had sensitive and hypersensitive skin; (4) had moles, acne, telangiectasia, etc., at the skin under study; (5) had used similar cosmetics or took antibiotics within 3 months; (6) had chronic diseases (asthma, diabetes mellitus, hypertension, etc.); (7) had atopic dermatitis. A total of 30 healthy Korean female volunteers (mean age of 43.8 and ranging in age from 26 to 53 years) participated in this study, and the participants were divided into two groups, high hydration group (HHG; *n* = 16, ≥50 A.U., arbitrary units) and low hydration group (LHG; *n* = 14, <50 A.U.), according to the hydration levels in their facial cheek skin. A set of basic cosmetics, consisting of skin softener (solubilized type), lotion (oil‐in‐water (O/W) emulsion type), essence (solubilized type), and cream (O/W emulsion type) containing moisturizing compounds was prepared in Coway Co. Ltd. (Korea, Tables S2 and S3) and sequentially applied twice a day (morning and evening) for 4 weeks (from June to July 2015) on their faces after facial washing with a cleanser (Coway, Korea). Measurements of skin biophysical parameters and swab sampling of facial cheek skin were performed three times (just before the use of the cosmetics and at 2 and 4 weeks after use of the cosmetics). Before the measurements of skin biophysical parameters and skin swab sampling, the participants relaxed for 20 min in a room with normal temperature and humidity (22 ± 2°C and 50% ± 5% relative humidity) after facial washing with a cleanser. All participants did not take antibiotics or steroids, and no other skin care products were applied to the skin regions under study the entire experiment.

### 2.2 Measurements of skin biophysical parameters

Skin biophysical parameters including skin hydration, TEWL, and roughness were measured at three different places of facial cheek skin. Briefly, skin hydration values were measured using a Corneometer CM825 instrument (Courage + Khazaka Electronic Gmbh, Germany) and expressed as arbitrary units (A.U.). Skin TEWL was measured with open‐chamber Tewameter TM300 (Courage + Khazaka Electronic Gmbh, Germany), according to the manufacturer's instructions. Facial skin roughness was analyzed using the three‐dimensional (3D) skin imaging system PRIMOS^®^ premium (GFMesstechnik GmbH, Germany). Skin roughness was evaluated using five roughness parameters (Ra; arithmetic average roughness, Rmax; maximum of all peak‐to‐valley values, Rz; average maximum height of the profile, Rp; maximum profile peak height, and Rv; maximum profile valley depth).

### 2.3 Skin swab sampling and amplification of 16S rRNA genes

For specimen sampling to analyze the bacterial community, facial cheek skin was swabbed with sterile swabs (Quick Swab, 3M Microbiology, USA), as described in Si et al. (2015), and the swab heads were stored at −80°C until use. The genomic DNA from the swab heads was extracted by a bead mill homogenization procedure, using a FastDNA Spin kit (MP Biomedicals, USA). Bacterial 16S rRNA genes containing hypervariable V1‐V3 regions, recommended for analysis of the skin microbiome community (Kong, 2016; Meisel et al., 2016), were PCR‐amplified using primer sets, Bac9F/Bac541R with 7–11 unique barcode sequences, as described previously (Lee et al., 2012). A composite sample was prepared by pooling equal amounts of PCR amplicons from each sample, and pyrosequencing was performed using a 454 GS FLX Titanium Sequencing System (Roche, Germany) at Chunlab (Korea).

### 2.4 Data analysis

Pyrosequencing data processing and analysis were carried out following the RDP pyrosequencing pipeline at http://pyro.cme.msu.edu (Cole et al., 2009). Pyrosequencing reads were sorted according to their barcode sequences to obtain sequences from each specimen sample, and then, the barcode sequences were removed. Pyrosequencing reads with less than two “N” residues (undetermined nucleotides) and read lengths longer than 300 nucleotides were selected for further analysis. Potential chimeric sequences were removed using UCHIME Chimera Slayer (Edgar, Haas, Clemente, Quince, & Knight, 2011) in the RDPipeline, and nonbacterial reads including chloroplast, mitochondrial, archaeal, and eukaryotic sequences were also discarded by the remove.lineage command within the Mothur program (Schloss et al., 2009). To compare bacterial diversity among specimen samples, the high‐quality sequencing reads were normalized to the smallest read number by random removal using the sub.sample command of the Mothur program. Operational taxonomic units (OTUs), Shannon & Weaver (1963) and Chao (1987) biodiversity indices, and evenness for the normalized sequencing reads were calculated with the RDPipeline at a 97% identity level. Bray–Curtis dissimilarity was evaluated using the Vegan package (Oksanen et al., 2015) of R (http://cran.r-project.org).

Taxonomic assignment of the high‐quality sequencing reads was performed using the RDP classifier (Wang, Garrity, Tiedje, & Cole, 2007) with an 80% confidence threshold at the phylum and genus levels. For the comparison of bacterial communities between specimen samples, the relative abundance data of bacterial communities at the genus level were input into MATLAB PLS_Toolbox (ver. 3.5, Eigenvector Research, USA) and mean‐centered with no scaling, and principal component analysis (PCA) and clustering analysis were performed.

Metabolic functions of the facial skin microbiome based on the bacterial 16S rRNA gene sequences were predicted by Phylogenetic Investigation of Communities by Reconstruction of Unobserved States (PICRUSt) (Langille et al., 2013). Briefly, a BIOM‐formatted OTU table with OTUs assigned to a Greengenes OTU ID at 97% identity was generated using the make.biom command of the Mothur program based on a greengenes database (May 2013 ver.; http://greengenes.lbl.gov). The resulting OTU table was uploaded into PICRUSt (ver. 1.0.0) on the web‐based Galaxy (http://huttenhower.sph.harvard.edu/galaxy) and relative abundance of Kyoto Encyclopedia of Genes and Genomes (KEGG) metabolic pathways at level 2 derived from the PICRUSt analysis was represented.

### 2.5 Statistical analysis

Paired or unpaired t‐tests were performed to determine statistical significance using SigmaPlot (ver. 10.0; Jandel Scientific, USA). The linear discriminant analysis (LDA) effective size (LEfSe) algorithm (Segata et al., 2011) was used to identify significantly different genus or KEGG pathway abundance between HHG and LHG or between before and after cosmetic use. The tables containing relative abundance of genera or KEGG metabolic pathways were imported into LEfSe (ver. 1.0) on the web‐based Galaxy, and only genera or KEGG metabolic pathways with logarithmic LDA scores >3.0 were included. Boxplots were constructed using the package “ggplot2” (Wickham, 2009) of R program.

### 2.6 Nucleotide sequence accession numbers

The pyrosequencing data of bacterial 16S rRNA genes are publicly available in the NCBI Short Read Archive under accession no. SRP090974 (NCBI BioProject PRJNA345237).

## 3 RESULTS

### 3.1 Skin hydration, TEWL, and roughness in the facial cheek skin of the high and low hydration groups

**Figure 1:**
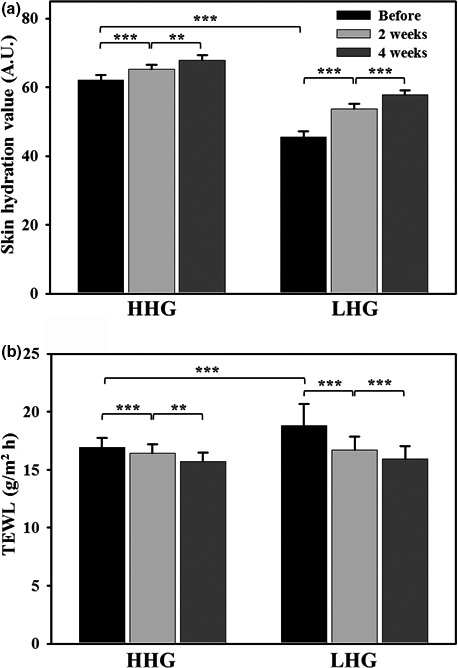
Changes in skin hydration value (a) and transepidermal water loss (b) following cosmetic use in facial cheek skin of the high hydration group (HHG) and low hydration group (LHG). Data in bar graphs are presented as means ± standard error, and the significance of differences is indicated by *p < .05; **,*p* < .01; and ***p < 0.001. A.U., arbitrary units; TEWL, transepidermal water loss

**Figure 2:**
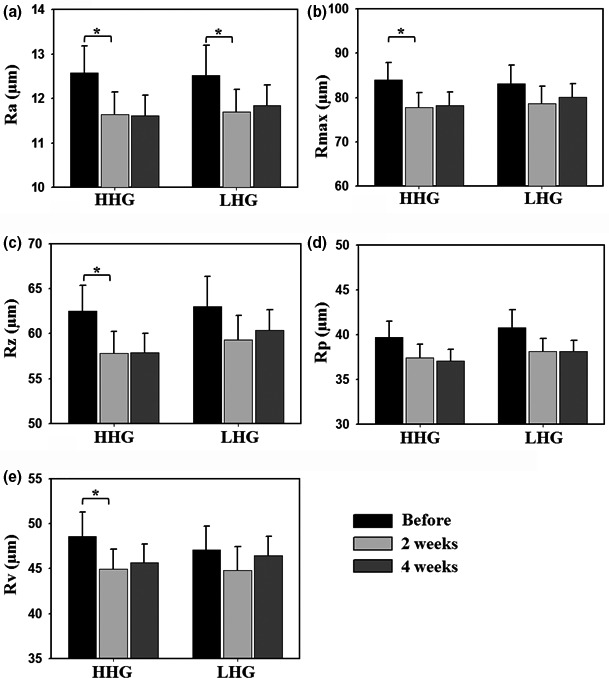
Changes in skin roughness following cosmetic use in facial cheek skin of the high hydration group (HHG) and low hydration group (LHG). Skin roughness parameters: *R*_a_, arithmetic mean roughness (a); *R*_max_, maximum roughness (b); *R*_z_, mean depth roughness (c); *R*_p_, maximum profile peak height (d); *R*_v_, maximum profile valley depth (e). Data in bar graphs are presented as means ± standard error, and the significance of differences is indicated by *p < .05

The individual participants were divided into two groups, HHG and LHG, based on the hydration level of 50 A.U. (arbitrary units) criterion and skin biophysical parameters and bacterial communities in HHG were used as a kind of normal skin control because the skin with more than 50 A.U. has been typically used as a standard skin control “sufficiently hydrated” in previous reported literatures and guidelines (Kwiatkowska, Franklin, Hendriks, & Kwiatkowski, 2009; Na et al., 2010; Van Kuilenburg, Masen, Groenendijk, Bana, & Van Der Heide, 2012). Participants belonging to either HHG or LHG used a set of the basic cosmetics for 4 weeks, and biophysical parameters including skin hydration, TEWL, and roughness in facial cheek skin were measured (Figures 1 and 2). Skin hydration levels in HHG and LHG were 62.1 ± 1.5 A.U. 45.5 ± 1.8 A.U., respectively (Figure 1a). With the use of basic cosmetics, the skin hydration levels increased significantly (by paired *t*‐test) and gradually to 67.7 ± 1.5 A.U. and 57.8 ± 1.3 A.U. in HHG and LHG, respectively, after 4 weeks (Figure 1a). In contrast to the skin hydration levels, TEWL values in facial cheek skin were significantly lower in HHG (16.9 ± 0.8 g/m^2^h) than in LHG (18.8 ± 1.9 g/m^2^h; unpaired *t*‐test, *p* < .001) (Figure 1b). Significant decreases (by paired *t*‐test) in TEWL values in HHG and LHG were observed after the use of the basic cosmetics, and eventually, after 4 weeks, the TEWL values of LHG (15.9 ± 0.8 g/m^2^h) became similar to those of HHG (15.7 ± 0.8 g/m^2^h; Figure 1b). Eventually, after use of the basic cosmetics for 4 weeks, skin hydration level and TEWL were not significantly different between HHG and LHG, although they were significantly different between HHG and LHG before cosmetic use. The values of skin biophysical parameters (*R*_a_, *R*_max_, *R*_z_, *R*_p_, and *R*v) representing skin roughness were similar in both groups although the skin hydration levels were significantly different (Figure 2). With the use of the basic cosmetics, the skin roughness was slightly improved for both groups in comparison with the value before cosmetic use.

### 3.2 Bacterial communities in the facial cheek skin of HHG and LHG

**Figure 3:**
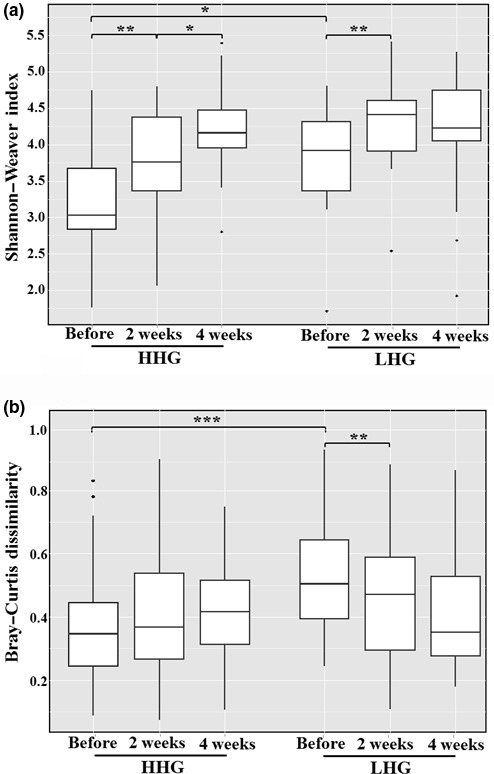
Box‐plots of Shannon–Weaver index (a) and Bray–Curtis dissimilarity (b) of bacterial 16S rRNA gene sequencing reads derived from the high hydration group (HHG) and low hydration group (LHG). The significance of differences between sampling groups is indicated by *p < .05; **p < 0.01; and ***p < .001

In total, 1,253,852 sequencing reads were generated by barcoded pyrosequencing of ninety specimen samples derived from the facial cheek skin of 30 participants. After the removal of low quality and nonbacterial reads, 721,446 bacterial 16S rRNA sequencing reads with high quality (57.54% of the total reads) were obtained for further analysis (Table S1). Because statistical diversity indices such as OTU and Shannon–Weaver and Chao1 indices are affected by the number of sequencing reads used, the diversity indices were calculated using normalized 16S rRNA gene sequences with 3,184 reads in each specimen sample. Shannon–Weaver and Chao1 indices and OTU, representing common measures of bacterial diversity in the facial cheek skin (i.e., alpha diversity), were generally greater in LHG than HHG (Table S1). Figure 3a shows that the Shannon–Weaver index, a representative alpha diversity index, was significantly greater in LHG than HHG (unpaired *t*‐test, *p* < .05). The Shannon–Weaver index increased with statistical significance after use of the cosmetics in both groups, especially in HHG (paired *t*‐test, *p* < .01), suggesting that the use of the basic cosmetics increased microbial diversity in facial skin. The Chao1 index, representing total bacterial species richness in each specimen sample, also increased slightly with the use of the cosmetics in both groups (Figure S1). The analysis of Bray–Curtis dissimilarity, representing the dissimilarity of bacterial composition between specimen samples (i.e., beta diversity), indicated that the dissimilarity was significantly higher in LHG than in HHG (unpaired *t*‐test, *p* < .001) before use of the basic cosmetics (Figure 3b). With use of the cosmetics, the Bray–Curtis dissimilarity values in HHG gradually increased, whereas the values in LHG gradually decreased, indicating that bacterial community compositions among specimen samples became more different in HHG, whereas they became more similar in LHG. In conclusion, the Shannon–Weaver index and Bray–Curtis dissimilarity values were significantly different between HHG and LHG before use of the cosmetics, but significant differences were not found after use of the basic cosmetics.

To compare the bacterial community in the facial cheek skin of HHG and LHG and to investigate the effects of the basic cosmetics on the facial cheek skin microbiome, bacterial 16S rRNA gene sequences were classified at the phylum and genus levels (Figures S2 and S3). Although the relative abundance of each bacterial group in each specimen sample was highly variable depending on the participants and specimen samples, the sequencing reads at the phylum level were predominantly affiliated with four phyla: *Actinobacteria* (5.2%–97.2%), *Proteobacteria* (1.5%–86.2%), *Firmicutes* (0.8%–54.2%), or *Bacteroidetes* (0.1%–33.4%) (Figure S2), which together accounted for at least 90.0% of total sequencing reads in all samples. Twenty‐seven other phylum members were also identified as minor groups from the facial cheek skin samples.

Sequencing reads of 734 genera at the genus level were found from the facial cheek skin samples, but only seven genera, *Propionibacterium* and *Corynebacterium* of the phylum *Actinobacteria*;* Ralstonia*,* Burkholderia*,* Cupriavidus*, and *Pelomonas* of the phylum *Proteobacteria*, and *Staphylococcus* of the phylum *Firmicutes*, were identified as common genera for all specimen samples (Figure S3). The relative abundance of each bacterial group at the genus level was also highly variable depending on the participants and specimen samples.

**Figure 4:**
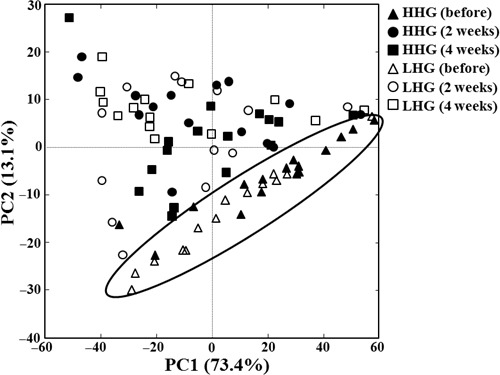
A principal component analysis score plot showing the change in bacterial communities after the use of cosmetics in facial cheek skin of the high hydration group (HHG) and low hydration group (LHG). PCA was performed using the relative abundance information at the genus level. The symbols in the ellipse represent bacterial communities in facial cheek skin of HHG and LHG just before the use of cosmetics

For statistical analysis of the bacterial communities in the facial cheek skin of HHG and LHG, PCA using the relative abundance of each bacterial group at the genus level (Figure S3) was performed (Figure 4). The PCA score plot showed that the symbols representing bacterial communities in the facial cheek skin of LHG before cosmetic use were more widely spread over the PC1 and PC2 regions than those of HHG, indicating that interpersonal variability in bacterial communities in LHG was higher than that in HHG, in accordance with the Bray–Curtis dissimilarity result of Figure 3b. After use of the cosmetics, the symbols were widespread over the PC1 and PC2 regions regardless of HHG or LHG, indicating that the effects of the cosmetics on skin bacterial communities were more dependent on individual participants rather than the skin hydration level. In addition, the symbols before cosmetics use were relatively linearly clustered, but after cosmetic use, the symbols were more widely scattered, indicating that the bacterial communities in facial skin after cosmetic use were different from those present before use of the cosmetics in both HHG and LHG.

**Figure 5:**
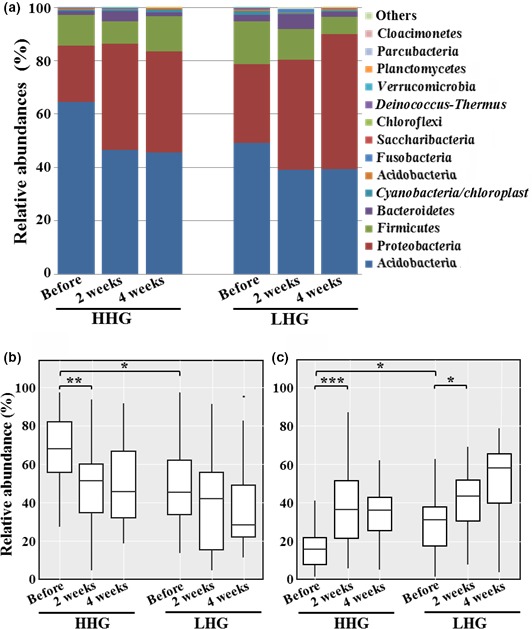
Mean abundances at the phylum level in facial cheek skin of high hydration group (HHG) and low hydration group (LHG) (a) and statistical box‐plot analysis for the relative abundance of *Actinobacteria* (b) and *Proteobacteria* (c). The mean abundances were the mean value of the relative abundance of each phylum in the sampling groups. Significant differences are indicated by *p < .05; **p < .01; and ***p < .001

**Figure 6:**
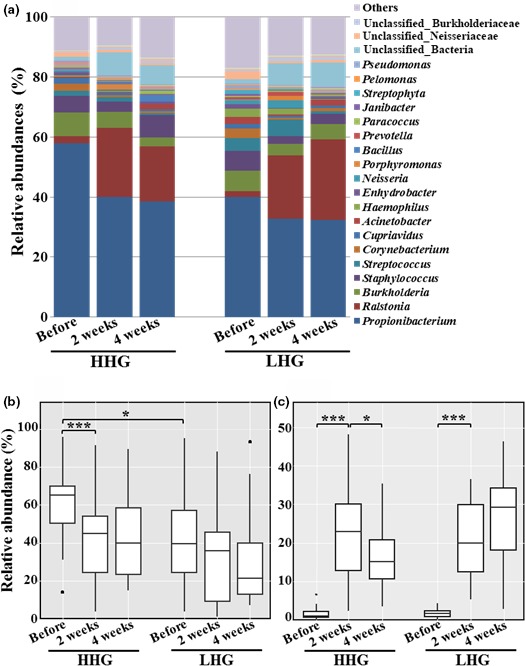
Mean abundances at the genus level in facial cheek skin of high hydration group (HHG) and low hydration group (LHG) (a) and statistical box‐plot analysis for the relative abundance of *Propionibacterium* (b) and *Ralstonia* (c). The mean abundance was the mean value of the relative abundance of each genus in the sampling groups, and significant differences are indicated by *p < .05 and ***p < .001

The interpersonal variability in bacterial communities was too high to allow drawing a clear conclusion on the effects of basic cosmetics on the facial skin microbiome. Therefore, bacterial communities within the sampling groups were investigated by assessing mean abundance of each bacterial population (Figures 5 and 6). At the phylum level, members of four phyla, *Actinobacteria*,* Proteobacteria*,* Firmicutes*, and *Bacteroidetes*, were predominant as described above, and their mean proportions accounted for more than 97.2% of the total bacterial population in all test groups (Figure 5a). Before use of the cosmetics, the relative abundance of *Actinobacteria* was higher in HHG than in LHG (unpaired *t*‐test, *p* < .05), whereas *Proteobacteria* were more abundant in LHG than in HHG (unpaired t‐test, *p* < .05) (Figure 5b and c). After cosmetic use, the relative abundance of *Actinobacteria* decreased in both HHG and LHG, especially in HHG, with statistical significance (paired *t*‐test, *p* < .01), whereas the relative abundance of *Proteobacteria* increased in both groups with statistical significance (Figure 5b and c). However, after cosmetic use, no significant differences were found in the relative abundance of *Actinobacteria* and *Proteobacteria* between HHG and LHG. In addition, the relative abundance of the other major phyla, *Firmicutes* and *Bacteroidetes*, was not significantly different between the hydration groups and in comparisons before and after cosmetic use (data not shown).

At the genus level, the genus *Propionibacterium* belonging to the phylum *Actinobacteria* was predominant in all test groups regardless of hydration level and cosmetic use, followed by the genera *Ralstonia*,* Burkholderia*,* Staphylococcus*,* Streptococcus*,* Corynebacterium*, and *Cupriavidus* (Figure 6a). Before cosmetic use, the relative abundance of *Propionibacterium* was significantly higher in HHG than in LHG (unpaired *t*‐test, *p* < .05) (Figure 6b). However, before cosmetic use, no other group among the major genus groups was significantly different between HHG and LHG, except for *Propionibacterium*. With cosmetic use, the relative abundance of *Propionibacterium* decreased in both groups, especially in HHG, with high statistical significance (paired *t*‐test, *p* < .001) (Figure 6b). In contrast, the relative abundance of *Ralstonia*, not previously reported to be part of the core human skin microbiome, increased in both groups with high statistical significance (paired *t*‐test, *p* < .001) (Figure 6c). However, no significant differences were found in the relative abundances of both *Propionibacterium* and *Ralstonia* between HHG and LHG after cosmetic use.

**Figure 7:**
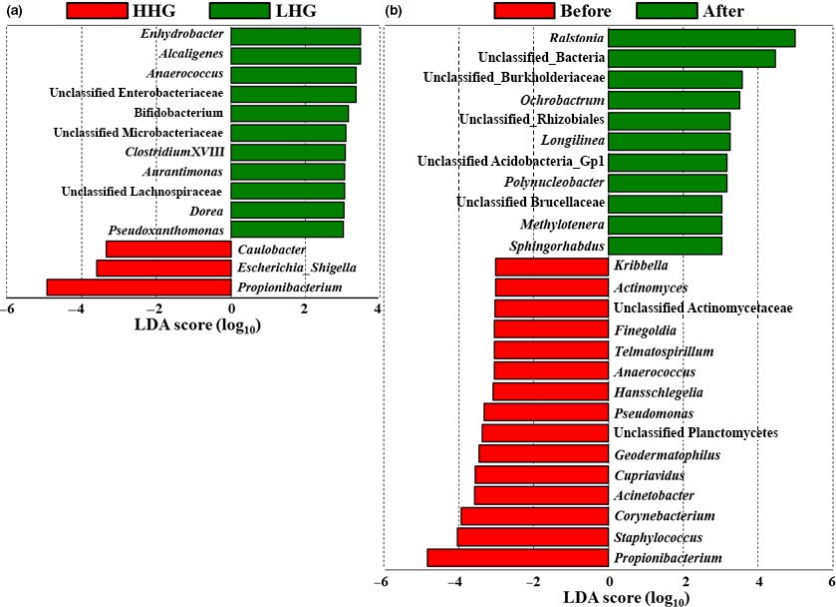
Linear discriminant analysis effect size (LEfSe) analysis of bacterial groups with differential abundance between the high hydration group (HHG) and low hydration group (LHG) before cosmetic use (a) and comparing abundance before and after cosmetic use regardless of HHG and LHG (b). Significance levels for LEfSe were *p* < .05, and only bacterial groups with a linear discriminant analysis (LDA) score >3 are displayed

The skin biophysical parameters except for skin roughness, bacterial diversity, and bacterial communities were obviously different between HHG and LHG before cosmetic use and in comparisons before and after cosmetic use. However, they were not significantly different between the hydration groups after cosmetic use. Therefore, additional analyses were limited to comparisons of bacterial populations between HHG and LHG before cosmetic use, or comparisons of bacterial populations before and after cosmetic use regardless of the hydration level. The linear discriminant analysis (LDA) effective size (LEfSe) analysis was performed to identify bacterial groups from all genera including minor genera with statistically differential abundance between HHG and LHG before cosmetic use, or before and after cosmetic use (Figure 7). The results clearly show that *Propionibacterium*, the most predominant bacterial genus, was more abundant in HHG than in LHG, but there were no differences in the other major bacterial genera of the facial skin microbiome between the hydration groups before cosmetic use (Figure 7a). The LEfSe analysis shows that among minor bacterial groups, *Escherichia*_*Shigella* and *Caulobacter* were more abundant in HHG than in LHG, whereas *Enhydrobacter, Alcaligenes, Anaerococcus,* unclassified *Enterobacteriaceae, Bifidobacterium,* unclassified *Microbacteriaceae, ClostridiumXVIII, Aurantimonas,* unclassified *Lachnospiraceae, Dorea*, and *Pseudoxanthomonas* were more abundant in LHG than in HHG (Figure 7a). The LEfSe analysis comparing population abundance before and after cosmetic use clearly shows that with cosmetic use the relative abundance of *Propionibacterium* in the facial skin microbiome significantly decreased, whereas that *Ralstonia* significantly increased (Figure 7b), as shown in Figure 6. For other bacterial groups, the relative abundance of *Staphylococcus*,* Corynebacterium*,* Acinetobacter*,* Cupriavidus*,* Geodermatophilus*, unclassified_*Planctomycetes*,* Pseudomonas*,* Hansschlegelia*,* Anaerococcus*,* Telmatospirillum*,* Finegoldia*, unclassified_*Actinomycetaceae*,* Actinomyces*, and *Kribbella* decreased after cosmetic use, whereas the relative abundance of unclassified_*Bacteria*, unclassified_*Burkholderiaceae*,* Ochrobactrum*, unclassified_*Rhizobiales*, unclassified_*Acidobacteria*_Gp1, *Longilinea*, unclassified_*Brucellaceae*,* Polynucleobacter*,* Methylotenera*, and *Sphingorhabdus* increased after use of the basic cosmetics (Figure 7b).

### 3.3 Prediction of metabolic functions of the skin microbiome by PICRUSt

**Figure 8:**
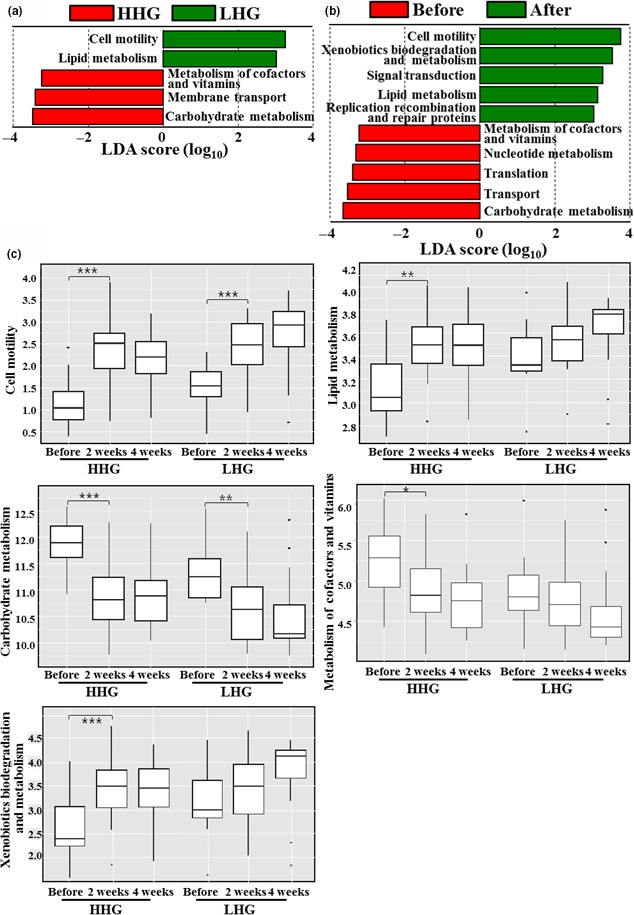
Linear discriminant analysis effect size (LEfSe) analysis of KEGG pathways with differential abundance between the high hydration group (HHG) and low hydration group (LHG) before cosmetic use (a) and comparing pathway abundance before and after cosmetic use regardless of HHG and LHG (b). Significance levels for LEfSe were *p* < .05, and only KEGG pathways with linear discriminant analysis (LDA) score >3 are displayed. Additional box‐plot analysis was performed for the representative KEGG pathways showing differential abundance to investigate the abundance change in KEGG pathways in response to cosmetic use in each hydration group (c). Significant differences are indicated by *p < .05; **p < .01; and ***p < 0.001

To predict metabolic functions of the facial cheek skin microbiome, a Phylogenetic Investigation of Communities by Reconstruction of Unobserved States (PICRUSt), a method that has been verified for the skin microbiome, was performed based on 16S rRNA gene sequences (Meisel et al., 2016). The relative abundance of KEGG categories representing metabolic functions of the facial skin microbiome is presented (Figure S4). KEGG categories related to membrane transport, replication and repair, amino acid metabolism, carbohydrate metabolism, and energy metabolism were predicted to be major metabolic functions of the facial cheek skin microbiome (>5% of relative abundance). LEfSe analysis based on the predicted KEGG categories derived from the PICRUSt analysis of the facial skin microbiome was carried out to identify differential metabolic functions between HHG and LHG before cosmetic use, or that differed before and after cosmetic use. From the results, carbohydrate metabolism, membrane transport, and metabolism of cofactors and vitamins were predicted to be more prominent KEGG categories in HHG than in LHG, whereas cell motility and lipid metabolism were predicted as more abundant KEGG categories in LHG than in HHG (Figure 8a). The LEfSe analysis of metabolic functions before and after cosmetic use showed that carbohydrate metabolism, transport, translation, nucleotide metabolism, and metabolism of cofactors and vitamins were predicted as more prominent KEGG categories before cosmetic use, whereas cell motility, xenobiotics biodegradation and metabolism, signal transduction, lipid metabolism, and replication recombination and repair proteins were predicted as more enriched KEGG categories after cosmetic use (Figure 8b). After cosmetic use, the abundance of the KEGG categories including cell motility, lipid metabolism, carbohydrate metabolism, metabolism of cofactors and vitamins, and xenobiotics biodegradation and metabolism were changed to an extent that was statistically significant. However, no significant differences in KEGG categories were observed between HHG and LHG after cosmetic use (Figure 8c), as was the case for skin biophysical parameters, bacterial diversity, and bacterial communities.

## 4 DISCUSSION

Skin hydration is one of the most important factors affecting the biophysical properties and functions of human skin, and an adequate level of skin hydration is critical for maintaining healthy skin (Wertz, 1996). Skin hydration is also an important environmental factor enabling colonization by microorganisms in human skin. Although microbial communities may be different depending on the skin hydration level, no extensive study on the relationships between skin hydration levels and microbial communities in human skin has been reported. Instead, some survey studies on skin microbiomes have been performed (Grice et al., 2009; Nakatsuji et al., 2013). Various skin problems, aging, and diseases occur more frequently and severely in dry skin than in normal skin, and the use of basic cosmetics has been suggested as a way to combat dry skin because moisturizing to increase the skin water hydration is one of the most important functions of basic cosmetics (Camargo, Gaspar, & Maia Campos, 2011; Chang, Huang, Chang, & Chang, 2008; Cheng et al., 2009). We hypothesized here that the use of basic cosmetics on dry skin might restore skin biophysical parameters, including hydration level, as well as change microbial communities in dry skin to resemble those in normal skin. Therefore, we decided to compare bacterial communities in the facial skin of HHG and LHG, and to measure skin biophysical parameters, in order to investigate the effects of basic cosmetics. To the best of our knowledge, there was no study investigating the effects of basic cosmetics on the skin microbiome.

### 4.1 Bacterial communities in facial skin

Previous studies have shown that *Actinobacteria*,* Firmicutes*,* Proteobacteria*, and *Bacteriodetes* are common and dominant bacterial phyla identified from human skin (Grice et al., 2009; Grice & Segre, 2011; The Human Microbiome Project Consortium, 2012; Grice, 2015). Our study using pyrosequencing also showed that these four phyla were common and dominant bacterial groups in facial cheek skin (Figure 5). Very diverse bacterial groups comprising as many as 734 genera were identified from the facial cheek skin. However, only seven genera (*Propionibacterium*,* Corynebacterium*,* Ralstonia*,* Burkholderia*,* Cupriavidus*,* Pelomonas*, and *Staphylococcus*), accounting for <1% of the total number of bacterial genera identified from the facial cheek skin, were identified as common bacterial groups in all participants (Figure S3). This was very low in comparison to a previous study stating that 6.6% of total genera were identified as common bacterial groups in forearm skin (Gao, Tseng, Pei, & Blaser, 2007). In particular, *Propionibacterium*, known as a very important lipophilic skin bacterial group, had a relatively high abundance of 32.3%–57.8% for the total facial bacteria (Figure 6), which might be caused by the secretion of high amounts of sebum in the facial skin compared to other skin areas (Costello et al., 2009; Gri Grice & Segre, 2011; Gribbon, Cunliffe, & Holland, 1993; Krutmann, 2009; Zeeuwen et al., 2012). The genera *Ralstonia*,* Burkholderia*, and *Cupriavidus* belonging to the phylum *Proteobacteria*, which have not been reported as common human skin bacterial genera (Cosseau et al., 2016; Fierer et al., 2008; Gao et al., 2007; Grice et al., 2008; Human Microbiome Project Consortium, 2012; Nakatsuji et al., 2013), were found to be dominant and common genera from the facial cheek skin in this study. However, previous studies reported that the order *Burkholderiales* including the genera *Ralstonia*,* Burkholderia*, and *Cupriavidus* was identified as a major bacterial group from the subepidermal compartments of facial skin and the superficial samples of forearm skin (Gao et al., 2007; Grice et al., 2008; Nakatsuji et al., 2013), suggesting that they may play an important role as common bacterial flora in human skin and further studies are necessary to understand more clearly their functional and physiological roles in human skin. High interpersonal variability in the skin bacterial microbiome has been reported in a previous study (Schommer & Gallo, 2013). Our study also showed that there was high interpersonal variability in bacterial community composition between participants, which may be caused by the direct exposure of skin to various different environments.

### 4.2 High hydration versus low hydration

We compared skin biophysical parameters and bacterial communities in facial cheek skins with different hydration levels (HHG and LHG). TEWL in HHG was lower than that in LHG (Figure 1), in accordance with a previous study (Mahrhauser, Nagelreiter, Baierl, Skipiol, & Valenta, 2015). Facial skin roughness was not significantly different depending on the skin hydration level (Figure 2). However, in previous studies (Cook & Craft, 1985; Linde, Bengtsson, & Loden, 1989), skin roughness in atopic skin or in dry skin that was clinically judged was significantly higher than that in normal skin. These results suggest that the division of LHG and HHG based on the hydration level in this study is different from that of dry skin and normal skin based on clinical judgment. Bray–Curtis dissimilarity analysis and PCA showed that the interpersonal variability in the bacterial community was higher in LHG than in HHG (Figures 3B and 4), in accordance with previous studies that dry skin had less diverse core microbiomes than moist skin did (Gao et al., 2007; Mathieu, Vogel, & Simonet, 2014), indicating that environmental conditions in the facial skin of LHG test participants are more diverse than in the facial skin of HHG individuals.

*Propionibacterium* and *Corynebacterium* belonging to *Actinobacteria*, and *Staphylococcus* belonging to *Firmicutes* have been reported to vary widely depending on skin hydration level and sebum content in human skin. The abundance of *Propionibacterium* is more related to sebum content, whereas the abundance of *Staphylococcus* and *Corynebacterium* is more strongly affected by moisture content (Costello et al., 2009; Gri Grice & Segre, 2011; Grice, 2015; Grice et al., 2009; Human Microbiome Project Consortium, 2012; Zeeuwen et al., 2012). However, among common and major bacterial genera in facial cheek skin, only the relative abundance of *Propionibacterium* was significantly different between HHG and LHG with high interpersonal variability (Figure 7A and Figure S3). The genus *Propionibacterium*, including *Propionibacterium acnes* known to be associated with acne, was significantly more abundant in HHG than in LHG (Figures 6b and 7a), probably suggesting that acne development may occur more easily in facial skin with a high hydration level. However, Fitz‐Gibbon et al. (2013) reported that certain strains of *P. acnes* were highly associated with acne development in skin, whereas other *P. acnes* strains were enriched even in healthy skin, suggesting that the physiological properties of *P. acnes* may be different depending on *P. acnes* strains and further studies about the roles of *P. acnes* in the skin at the strain level are necessary. The relative abundance of *Staphylococcus* and *Corynebacterium* was not significantly different between HHG and LHG. In particular, the genus *Staphylococcus*, including *Staphylococcus epidermis* known as a natural (beneficial) component of skin microflora (Krutmann, 2009), was similar in HHG and in LHG. These results (no difference in facial skin roughness and *Staphylococcus* abundance between HHG and LHG, and higher abundance of *Propionibacterium* in HHG than in LHG) suggest that we may need to change previous dichotomous viewpoints of high skin hydration as good in comparison with low skin hydration, and that *Propionibacterium* is harmful, and *Staphylococcus* beneficial.

The LEfSe analysis of the PICRUSt data showed that lipid metabolism and cell motility were predicted to be more abundant KEGG categories in LHG than in HHG, whereas carbohydrate metabolism, membrane transport, and metabolism of cofactors and vitamins were predicted to be more abundant KEGG categories in HHG than in LHG (Figure 8a). These results may stem from functional genes related to lipid metabolism and cell motility being unexpectedly less abundant in genomes of *Propionibacterium* compared to those of other skin bacteria, although *Propionibacterium* has a high triglyceride metabolic ability. A previous study reported that dry skin secreted higher amounts of sebum compared to normal skin (Youn, Kim, Hwang, & Park, 2002), which may well explain the higher abundance of lipid metabolism in LHG than in HHG.

### 4.3 Effects of basic cosmetics on the facial cheek skin microbiome

As reported in many previous studies (Camargo et al., 2011; Leite e Silva et al., 2009), increased skin hydration values and decreased TEWL and skin roughness were observed for facial cheek skin after cosmetic use in both HHG and LHG (Figures 1 and 2). The bacterial diversity increased significantly in both HHG and LHG with use of the basic cosmetics (Figure 3A), suggesting that the use of cosmetics might cause an increase in bacterial diversity by the input of diverse cosmetic components into the facial skin.

The relative abundance of typical skin bacterial groups including *Propionibacterium*,* Staphylococcus*, and *Corynebacterium* decreased after use of the basic cosmetics (Figures 6 and 7B), which might be due to growth of other skin bacteria utilizing components of the basic cosmetics, or inhibition by the cosmetics of growth of the typical skin bacterial groups, or changed environmental conditions. Interestingly, after use of the basic cosmetics, we observed a statistically significant decrease in *Propionibacterium*, known as a lipophilic and predominant resident in sebaceous environments, and a statistically significant increase in *Ralstonia*, not a core human skin bacterial group, in facial cheek skin regardless of HHG and LHG (Figures 6 and 7b). Although members of the genus *Propionibacterium* have the ability to metabolize triglycerides, they may not utilize the oil components of the basic cosmetics in skin. Instead of *Propionibacterium*, other bacteria such as *Ralstonia* may be able to metabolize the oil components of the basic cosmetics. Because a member of the genus *Propionibacterium*,* P. acnes*, is known to be associated with acne (Krutmann, 2009), the use of basic cosmetics may be helpful to diminish the development of acne in facial skin by decreasing *Propionibacterium*. Although the order *Burkholderiales* of the class *Betaproteobacteria* possibly including the genus *Ralstonia* was prominent in subepidermal compartments containing high lipid content (Nakatsuji et al., 2013), the dominance of *Ralstonia* in the human skin microbiome was not reported until now, suggesting that the dominance of *Ralstonia* in facial cheek skin can be used as an indicator for the use of basic cosmetics.

The functional and physiological properties of the genus *Ralstonia* in human skin have not been explored. Before use of the basic cosmetics, the relative abundance of *Ralstonia* was low regardless of the hydration level (Figure 6a and c). Therefore, the increase in the skin hydration level after the use of the basic cosmetics might not be an important reason for the increase in the prevalence of the genus *Ralstonia* after cosmetic use. The LEfSe analysis of KEGG categories derived from the PICRUSt data showed that predicted KEGG categories such as lipid metabolism and cell motility increased after use of the basic cosmetics (Figure 8). Members of *Ralstonia* have been reported to have the ability to metabolize various hydrocarbons including aliphatic, aromatic, and xenobiotic compounds (Ghosal, Ghosh, Dutta, & Ahn, 2016), which may suggest that the increased in the lipid metabolism, xenobiotics biodegradation and metabolism, and signaling transduction categories was related to the enrichment of *Ralstonia* in facial cheek skin after the use of the basic cosmetics.

In the LEfSe analysis, none of the bacterial genus groups or metabolic gene categories were identified as common to both facial cheek skin of HHG before cosmetic use and facial cheek skin after cosmetic use (Figures 7 and 8). In addition, the relative abundance of *Propionibacterium* and KEGG categories of carbohydrate metabolism, membrane transport, and metabolism of cofactors and vitamins that were statistically more abundant in facial skin of HHG than LHG before cosmetic use decreased in a statistically manner after cosmetic use. Metabolic gene categories (cell motility and lipid metabolism) that were more abundant in LHG than in HHG before cosmetic use were still statistically more abundant after cosmetic use than before cosmetic use, suggesting that skin bacterial communities of LHG did not shift to being similar to those of HHG after cosmetic use, although hydration levels and biophysical parameters of LHG were restored to resemble those of HHG by cosmetic use. These results suggest that bacterial communities in dry skin do not shift to those in normal skin just by the increase in skin hydration level using basic cosmetics and skin hydration level may not be a critical factor in determining the composition of skin microbial communities. Therefore, we need to investigate many other factors including cosmetic components, climate, and change in study conditions, particularly because preservatives such as methylparaben, one of the ingredients of cosmetics, have high influence on skin microbiome.

## CONFLICT OF INTEREST

None declared.
