# Supplementary material for: Effects of cosmetics on the skin microbiome of facial cheeks with different hydration levels
Source: Microbiologyopen. 2017 Nov 29;7(2):e00557. doi: 10.1002/mbo3.557 (PMC5911989; doi:10.1002/mbo3.557)
Supplement: Supplementary file 1 [file MBO3-7-na-s001.docx]

**Effects of Cosmetics on the Skin Microbiome of Facial Cheeks with Different Hydration Levels**

Hyo Jung Lee^1^, Sang Eun Jeong^2^, Soyoun Lee^3^, Sungwoo Kim^3^, Hyuntak Han^3^ and Che Ok Jeon^2,*^

^1^*Department of Biology, Kunsan National University, Gunsan 54150, Republic of Korea*

^2^*Department of Life Science, Chung-Ang University, Seoul* *06974, Republic of Korea*

^3^*Coway Cosmetics R&D Center, Seoul 08502, Republic of Korea*

*Corresponding author: Che Ok Jeon (E-mail: [cojeon@cau.ac.kr](mailto:cojeon@cau.ac.kr))

**Supplementary Figure 1.** Box-plot of Chao1 index of bacterial 16S rRNA gene sequencing reads derived from the high hydration group (HHG) and low hydration group (LHG). The sequencing was conducted just before the use of the basic cosmetics and at two and four weeks after the use of the basic cosmetics. Significant differences among sampling groups are indicated by *, P < 0.05 and ***, P < 0.001.


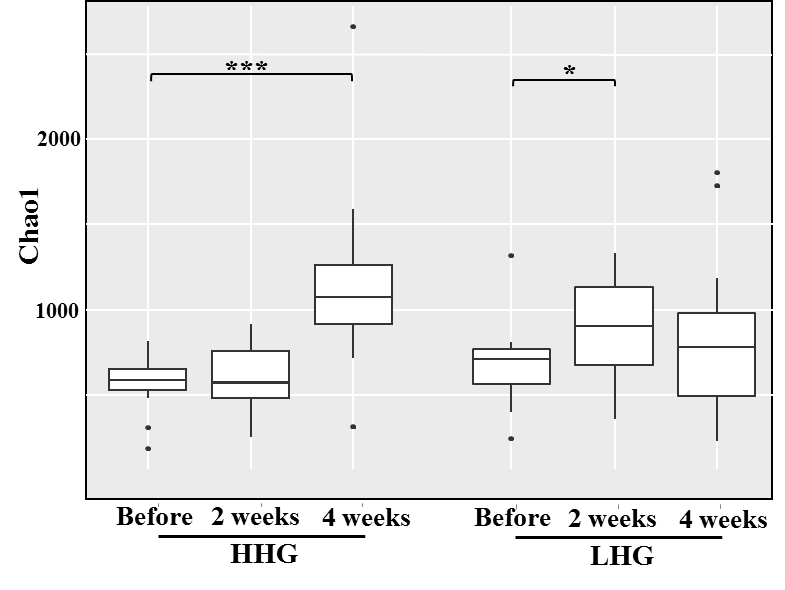


**Supplementary Figure 2.** Relative bacterial compositions of the specimen samples derived from facial cheek skin of the high hydration group (a, 16 participants) and low hydration group (b, 14 participants) at the phylum level. The analyses were conducted just before the use of the basic cosmetics and at two and four weeks after the use of the basic cosmetics. The 16S rRNA gene sequences were classified using the RDP classifier at an 80% confidence threshold.


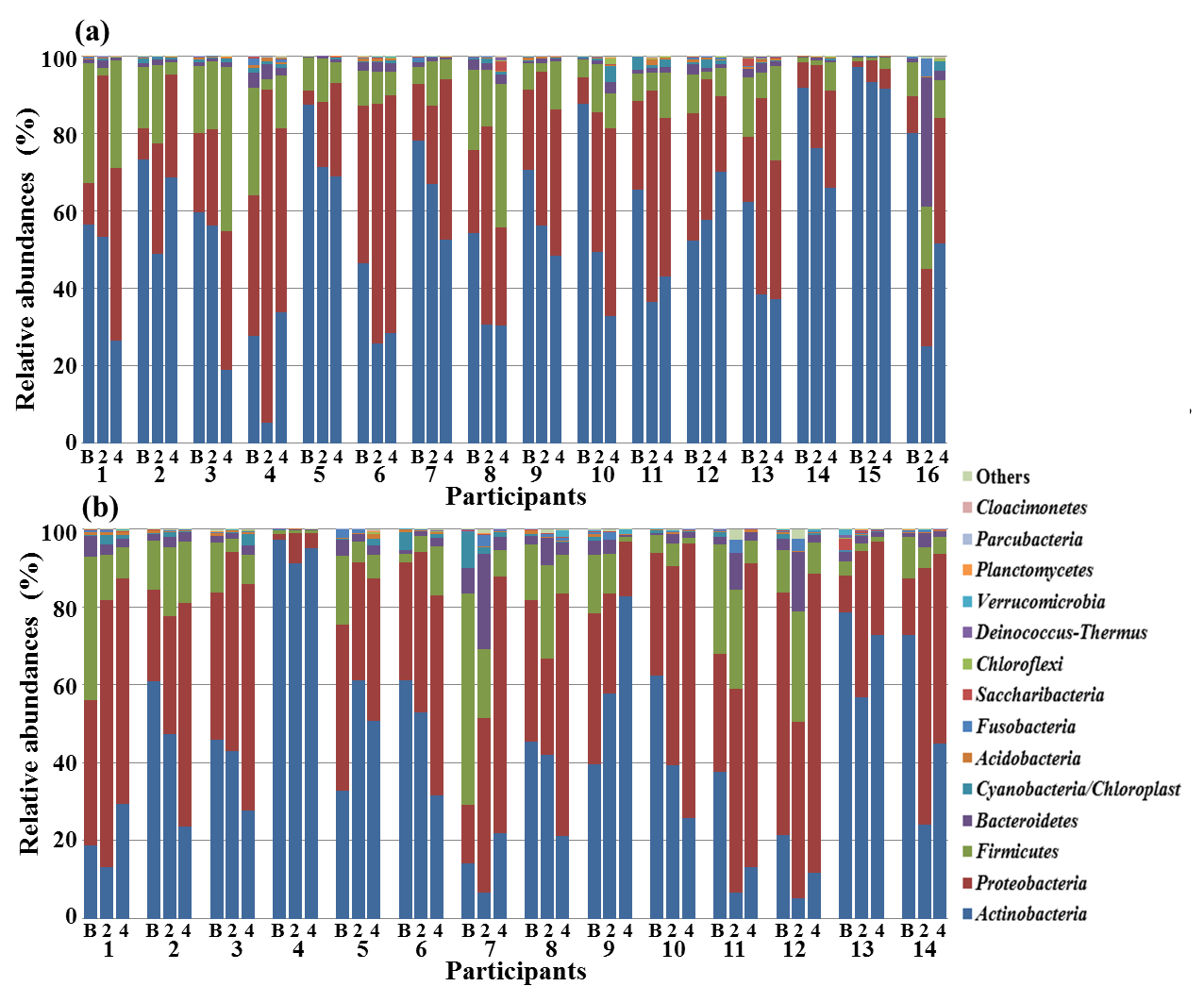


**Supplementary Figure 3.** Relative bacterial compositions of the specimen samples derived from facial cheek skin of the high hydration group (a, 16 participants) and low hydration group (b, 14 participants) at the genus level. The analyses were conducted just before the use of the basic cosmetics and at two and four weeks after the use of the basic cosmetics. The 16S rRNA gene sequences were classified using the RDP classifier at an 80% confidence threshold.


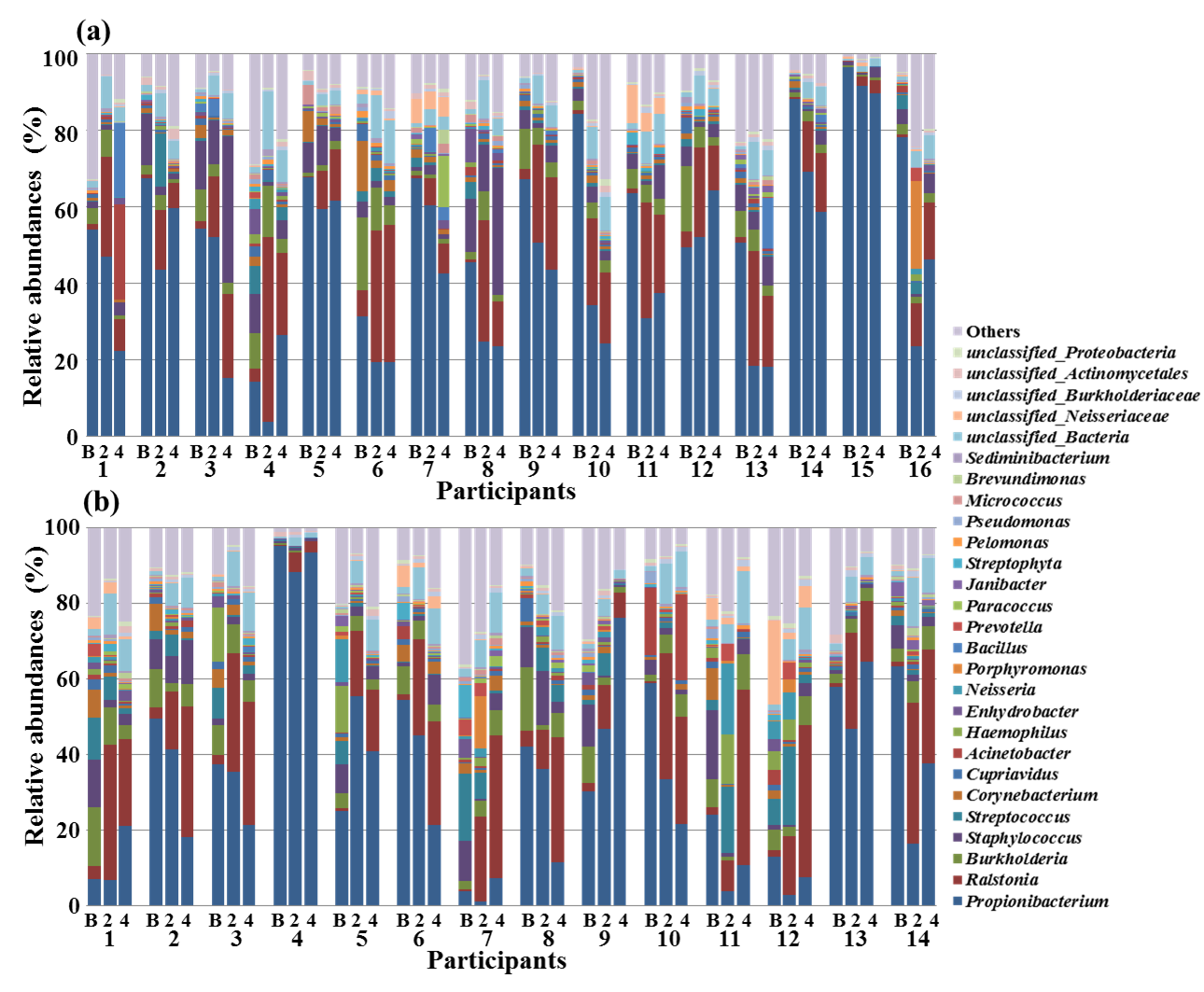


**Supplementary Figure 4.** PICRUSt analysis to predict metabolic functions of the facial cheek skin microbiome in the high hydration group (a) and low hydration group (b). The analyses were conducted just before the use of the basic cosmetics and at two and four weeks after the use of the basic cosmetics. The metabolic functions were compared using the relative abundance of KEGG pathways at level 2.


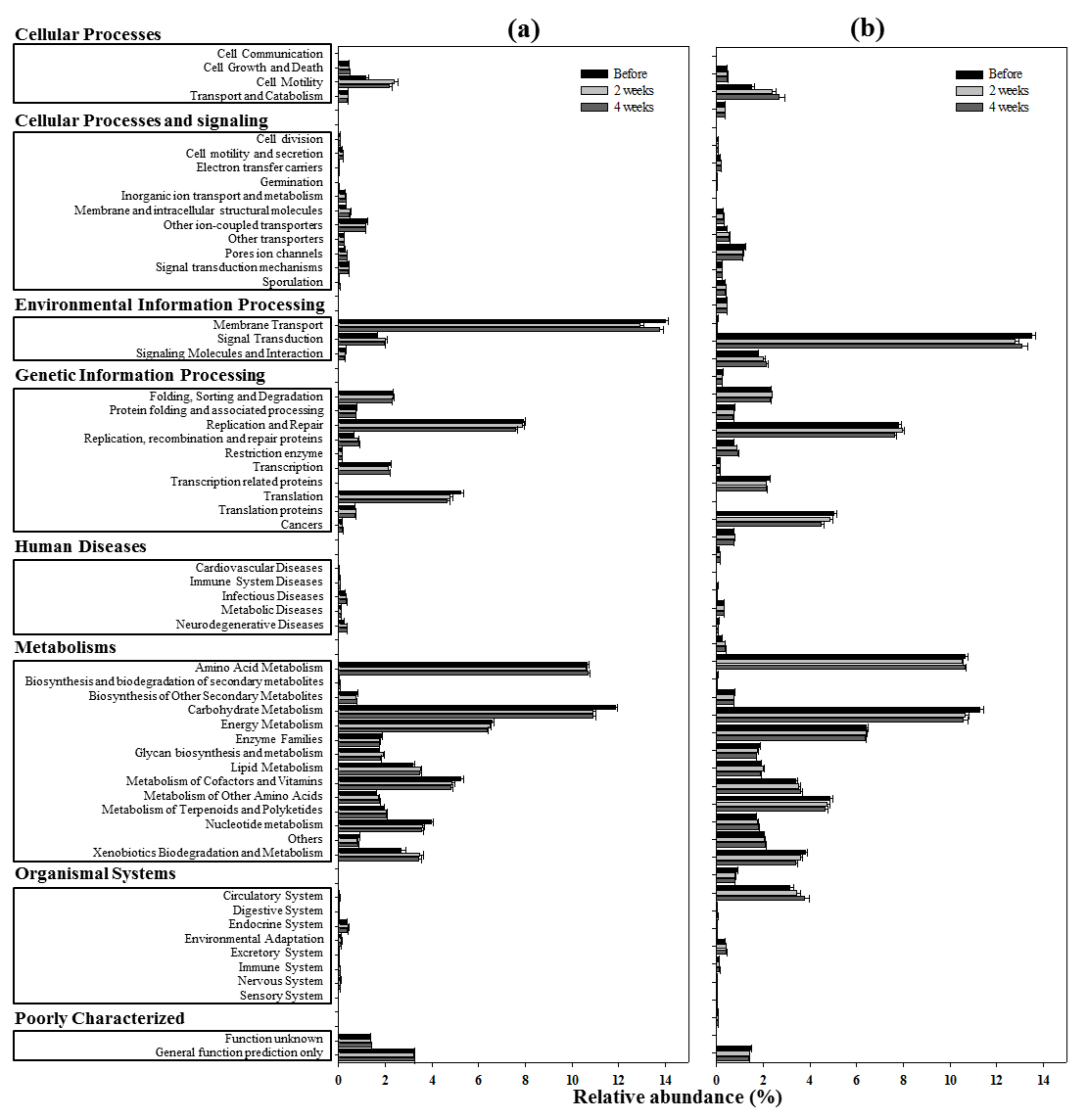


**Supplementary Table 1.** Pyrosequencing data and statistical analysis of bacterial 16S rRNA genes from the 30 specimen samples derived from facial cheek skin of the high hydration group (HHG 1–16) and low hydration group (LHG 1–14). The community analysis was conducted just before the use of the basic cosmetics and at two and four weeks after the use of the basic cosmetics.

| **Participant** | **Sampling time** | **No. of total reads** | **No. of high quality reads** | **OTUs^§^** | **Chao1^§^** | **Shannon-Weaver^§^** | **Evenness^§^** |
| --- | --- | --- | --- | --- | --- | --- | --- |
| HHG1 | Before use | 8609 | 5212 | 255 | 489 | 2.93 | 0.53 |
|  | 2 weeks | 10733 | 6784 | 274 | 451 | 3.31 | 0.59 |
|  | 4 weeks | 18241 | 10047 | 471 | 946 | 4.35 | 0.71 |
| HHG2 | Before use | 9476 | 5398 | 338 | 733 | 2.84 | 0.49 |
|  | 2 weeks | 9872 | 5992 | 354 | 647 | 3.75 | 0.64 |
|  | 4 weeks | 26740 | 15114 | 653 | 1307 | 4.47 | 0.69 |
| HHG3 | Before use | 8314 | 5407 | 345 | 651 | 3.37 | 0.58 |
|  | 2 weeks | 9250 | 5882 | 287 | 609 | 3.41 | 0.60 |
|  | 4 weeks | 9133 | 5192 | 468 | 1071 | 4.04 | 0.66 |
| HHG4 | Before use | 7153 | 5044 | 477 | 821 | 4.74 | 0.77 |
|  | 2 weeks | 9489 | 6438 | 309 | 493 | 3.76 | 0.66 |
|  | 4 weeks | 9600 | 5894 | 732 | 1594 | 5.22 | 0.79 |
| HHG5 | Before use | 8212 | 4991 | 293 | 604 | 3.03 | 0.53 |
|  | 2 weeks | 8425 | 5173 | 304 | 630 | 3.36 | 0.59 |
|  | 4 weeks | 22272 | 11865 | 417 | 828 | 3.68 | 0.61 |
| HHG6 | Before use | 7636 | 5298 | 350 | 669 | 3.79 | 0.65 |
|  | 2 weeks | 9632 | 6908 | 330 | 497 | 4.01 | 0.69 |
|  | 4 weeks | 13848 | 7933 | 542 | 1196 | 4.64 | 0.74 |
| HHG7 | Before use | 6285 | 3771 | 330 | 630 | 3.10 | 0.53 |
|  | 2 weeks | 18400 | 9349 | 409 | 837 | 3.92 | 0.65 |
|  | 4 weeks | 15262 | 8430 | 396 | 745 | 4.13 | 0.69 |
| HHG8 | Before use | 10032 | 6917 | 441 | 770 | 4.04 | 0.66 |
|  | 2 weeks | 19176 | 10436 | 315 | 466 | 4.16 | 0.72 |
|  | 4 weeks | 15511 | 8905 | 511 | 1133 | 4.39 | 0.70 |
| HHG9 | Before use | 11035 | 6470 | 272 | 544 | 2.83 | 0.50 |
|  | 2 weeks | 19954 | 9472 | 273 | 474 | 3.75 | 0.67 |
|  | 4 weeks | 9314 | 5234 | 447 | 977 | 4.28 | 0.70 |
| HHG10 | Before use | 8792 | 4851 | 254 | 569 | 2.30 | 0.42 |
|  | 2 weeks | 16300 | 9258 | 423 | 730 | 4.37 | 0.72 |
|  | 4 weeks | 17739 | 11361 | 1017 | 2659 | 5.39 | 0.78 |
| HHG11 | Before use | 10240 | 5875 | 263 | 561 | 2.85 | 0.51 |
|  | 2 weeks | 14262 | 7917 | 463 | 893 | 4.46 | 0.73 |
|  | 4 weeks | 28935 | 16983 | 461 | 1079 | 4.04 | 0.66 |
| HHG12 | Before use | 9703 | 6498 | 310 | 482 | 3.38 | 0.59 |
|  | 2 weeks | 9407 | 5806 | 267 | 546 | 3.25 | 0.58 |
|  | 4 weeks | 21735 | 10495 | 431 | 1034 | 3.95 | 0.65 |
| HHG13 | Before use | 8592 | 5721 | 370 | 605 | 3.67 | 0.62 |
|  | 2 weeks | 14707 | 8861 | 469 | 860 | 4.69 | 0.76 |
|  | 4 weeks | 14629 | 8694 | 571 | 1249 | 4.85 | 0.76 |
| HHG14 | Before use | 7490 | 3961 | 176 | 311 | 2.10 | 0.41 |
|  | 2 weeks | 10849 | 6104 | 273 | 520 | 2.98 | 0.53 |
|  | 4 weeks | 9278 | 5001 | 369 | 718 | 3.90 | 0.66 |
| HHG15 | Before use | 30750 | 13564 | 113 | 191 | 1.76 | 0.37 |
|  | 2 weeks | 9934 | 5115 | 146 | 253 | 2.06 | 0.41 |
|  | 4 weeks | 19246 | 8775 | 163 | 315 | 2.80 | 0.55 |
| HHG16 | Before use | 11908 | 6500 | 267 | 551 | 2.50 | 0.45 |
|  | 2 weeks | 10870 | 6489 | 479 | 918 | 4.80 | 0.78 |
|  | 4 weeks | 22730 | 13365 | 590 | 1385 | 4.16 | 0.65 |
| LHG1 | Before use | 4074 | 3184 | 473 | 660 | 4.81 | 0.78 |
|  | 2 weeks | 11472 | 7614 | 401 | 630 | 4.42 | 0.74 |
|  | 4 weeks | 13330 | 8079 | 786 | 1726 | 5.27 | 0.79 |
| LHG2 | Before use | 23201 | 13560 | 402 | 776 | 3.80 | 0.63 |
|  | 2 weeks | 15244 | 8573 | 525 | 1096 | 4.40 | 0.70 |
|  | 4 weeks | 21700 | 13635 | 412 | 833 | 4.06 | 0.67 |
| LHG3 | Before use | 10440 | 7015 | 361 | 637 | 4.03 | 0.68 |
|  | 2 weeks | 10081 | 6467 | 313 | 611 | 3.66 | 0.64 |
|  | 4 weeks | 21205 | 13165 | 544 | 1002 | 4.27 | 0.68 |
| LHG4 | Before use | 10195 | 4611 | 129 | 247 | 1.71 | 0.35 |
|  | 2 weeks | 18376 | 8526 | 166 | 360 | 2.54 | 0.50 |
|  | 4 weeks | 27197 | 11916 | 126 | 235 | 1.92 | 0.40 |
| LHG5 | Before use | 10543 | 7402 | 378 | 690 | 4.24 | 0.71 |
|  | 2 weeks | 18185 | 9472 | 399 | 756 | 3.88 | 0.65 |
|  | 4 weeks | 21063 | 12412 | 772 | 1807 | 4.93 | 0.74 |
| LHG6 | Before use | 9854 | 5844 | 285 | 437 | 3.30 | 0.58 |
|  | 2 weeks | 19966 | 10900 | 353 | 665 | 3.94 | 0.67 |
|  | 4 weeks | 13696 | 7720 | 640 | 1188 | 4.98 | 0.77 |
| LHG7 | Before use | 8401 | 5647 | 596 | 1323 | 4.81 | 0.75 |
|  | 2 weeks | 18564 | 11056 | 687 | 1336 | 5.41 | 0.83 |
|  | 4 weeks | 10097 | 6004 | 487 | 865 | 4.83 | 0.78 |
| LHG8 | Before use | 10789 | 7134 | 280 | 403 | 3.56 | 0.63 |
|  | 2 weeks | 15165 | 8397 | 556 | 1145 | 4.48 | 0.71 |
|  | 4 weeks | 21421 | 13748 | 486 | 910 | 4.47 | 0.72 |
| LHG9 | Before use | 8792 | 5756 | 424 | 739 | 4.16 | 0.69 |
|  | 2 weeks | 14720 | 8487 | 569 | 1096 | 4.54 | 0.72 |
|  | 4 weeks | 25951 | 12443 | 231 | 511 | 2.68 | 0.49 |
| LHG10 | Before use | 10387 | 5613 | 304 | 543 | 3.11 | 0.54 |
|  | 2 weeks | 15230 | 8909 | 371 | 724 | 4.09 | 0.69 |
|  | 4 weeks | 14732 | 8530 | 299 | 512 | 4.19 | 0.74 |
| LHG11 | Before use | 9279 | 6537 | 453 | 809 | 4.34 | 0.71 |
|  | 2 weeks | 10742 | 5893 | 638 | 1162 | 5.15 | 0.80 |
|  | 4 weeks | 12868 | 7219 | 305 | 469 | 4.27 | 0.75 |
| LHG12 | Before use | 8902 | 6274 | 524 | 813 | 4.72 | 0.75 |
|  | 2 weeks | 14486 | 8822 | 682 | 1288 | 5.34 | 0.82 |
|  | 4 weeks | 16784 | 10543 | 410 | 731 | 4.04 | 0.67 |
| LHG13 | Before use | 9230 | 5770 | 399 | 743 | 3.53 | 0.59 |
|  | 2 weeks | 13073 | 7300 | 372 | 782 | 3.89 | 0.66 |
|  | 4 weeks | 26396 | 14564 | 265 | 487 | 3.07 | 0.55 |
| LHG14 | Before use | 9762 | 5888 | 406 | 762 | 3.27 | 0.54 |
|  | 2 weeks | 12440 | 7025 | 524 | 1027 | 4.62 | 0.74 |
|  | 4 weeks | 16119 | 9042 | 315 | 492 | 4.11 | 0.71 |

^§^ Diversity indices of 16S rRNA gene sequencing reads in each sample were calculated using the normalized reads (3,184 reads) at a 97% sequence identity level.

**Supplementary Table 2.** Lists of ingredients of skin softener, lotion, essence, and cream used in this study.

| **Ingredient** | **Skin softener** | **Lotion** | **Essens** | **Cream** |
| --- | --- | --- | --- | --- |
| **Chemical component** | 2-Phenoxyethanol | 2-Phenoxyethanol | 1,2-Hexanediol | 1,2-Hexanediol |
|  | Acetyl Decapeptide-3 | Acetyl Decapeptide-3 | 2-Phenoxyethanol | Acrylates/C10-30 Alkyl Acrylate Crosspolymer |
|  | Acrylates/C10-30 Alkyl Acrylate Cross- polymer | Alcohol | Acetyl Decapeptide-3 | Adenosine |
|  | Alcohol | Aleurites Moluccana Seed Oil | Adenosine | Arginine |
|  | Benzophenone-5 | Betaine | Adenosine Triphosphate | Batyl Alcohol |
|  | Betaine | Bis-Methoxy PEG-40 Polyepsilon Caprolactone | Alcohol | Behenyl Alcohol |
|  | Biosaccharide Gum-1 | Butylene Glycol | Alginic Acid | Betaine, |
|  | Bis-Methoxy PEG-40 Polyepsilon Caprolactone | CAMELINA SATIVA SEED OIL | Beta-Glucan | Bis-PEG-18 Methyl Ether Dimethyl |
|  | Bis-PEG-18 Methyl Ether Dimethyl | EDTA-2Na | Biosaccharide Gum-1 | Butylene Glycol |
|  | Butylene Glycol | N-Acetylphytosphingosine | Bis-Methoxy PEG-40 Polyepsilon Caprolactone | Caprylhydroxamic Acid |
|  | Caprylic/Capric triglyceride | Oligopeptide-34 | Bis-PEG-18 Methyl Ether Dimethyl | Caprylic/Capric Triglyceride |
|  | Carbomer |  | Butylene Glycol | Caprylyl Glycol |
|  | Ceteareth-20 | Caprylic/Capric triglyceride | Caprylic/Capric triglyceride | Cetearyl Alcohol |
|  | Cetyl Alcohol, | Carbomer | Chlorphenesin | Cholesterol |
|  | Cholesterol | Ceteareth-20 | Cholesterol | Cholesteryl/Behenyl/Octyldodecyl Lauroyl Glutamate |
|  | Decapeptide 4 | Cetearyl Alcohol | Decapeptide 4 | Cyclopentasiloxane, Dimethicone / Vinyl Dimethicone Crosspolymer |
|  | Diisopropyl Adipate | Cetyl alcohol, | Diisopropyl Adipate | Dimethicone |
|  | Disodium Adenosine Triphosphate | Cholesterol, | Dipotassium Glycyrrhizate | Glycerin |
|  | Disodium Bis-Dioleoyl Glycerophosphoglycerin | Decapeptide 4 | Disodium Bis-Dioleoyl Glycerophosphoglycerin | Glycosphingolipids |
|  | EDTA-2Na | Dicaprylate/Dicaprate | Disodium Bis-Retinamido Methylpentane | Hydrogenated C6-14 Olefin Polymers |
|  | Ethanol | Diisopropyl Adipate | EDTA-2Na | Maltitol |
|  | Glycerin | Dipotassium Glycyrrhizate | Glycerin | Mangifera Indica (Mango) Seed Butter |
|  | Hydrogenated Lecithin | Disodium Adenosine Triphosphate | Hydrogenated Lecithin | Niacinamide |
|  | Hydrogenated Polydecene | Disodium Bis-Dioleoyl Glycerophosphoglycerin | Hydrogenated Polydecene | Panthenol |
|  | Hydrolyzed Oat Protein | Glycerin | Hydrolyzed Oat protein | Pentaerythrityl Tetraethylhexanoate |
|  | Lecithin | Glyceryl Stearate | Hydroxyethyl Urea | Phospholipids |
|  | Lecithin/Phosphatidylserine | Hydrogenated Lecithin | Niacinamide | Polyacrylate Crosspolymer-6 |
|  | Methyl Gluceth-20 | Hydrogenated Polydecene | Oligopeptide-24 | Propanediol |
|  | Methylparaben | Hydrolyzed Oat protein | Oligopeptide-34 | Sodium Hyaluronate |
|  | N-Acetylphytosphingosine | Lecithin | PEG-75 | Squalane |
|  | Nicotinamide | Lecithin/Phosphatidylserine | Polyphosphorylcholine Glycol Acrylate | Tromethamine |
|  | Octyldodeceth-16 | Limnanthes Alba (Meadowfoam) Seed Oil | Propandiol | Tropolone |
|  | Oligopeptide-24 | Methylparaben | Stearic Acid | Water |
|  | Oligopeptide-34 | Nicotinamide | Tocopheryl Acetate | Xanthan Gum |
|  | Panthenol | Oligopeptide-24 | Ubiquinone |  |
|  | PEG/PPG-17/6 Copolymer | Panthenol | Water |  |
|  | PEG-60 Hydrogenated Castor Oil | PEG-100 Stearate |  |  |
|  | PEG-8 | PEG-8 |  |  |
|  | Phenoxyethanol | Polyglyceryl-3 Methylglucose Distearate |  |  |
|  | Phenyl Trimethicone | Polyphosphorylcholine Glycol Acrylate |  |  |
|  | Polyphosphorylcholine Glycol Acrylate | Propylparaben |  |  |
|  | Sodium Acrylates/ Acrylonitrogens Copolymer | Sclerocarya Birrea Seed Oil |  |  |
|  | Sodium Hyaluronate | Sodium Hyaluronate |  |  |
|  | Stearic acid | Sorbitan stearate |  |  |
|  | Tetraacetylphytosphingosine | Stearic Acid |  |  |
|  | Tocopheryl Acetate | Tetraacetylphytosphingosine, |  |  |
|  | Triethanolamine | Tocopheryl Acetate |  |  |
|  | Ubiquinone | Ubiquinone |  |  |
|  | Water | Water |  |  |
| **Extract** | Jania rubens Extract | Jania rubens Extract | Commiphora myrrha leaf cell extract | Aquilaria agallocha Stem Extract |
|  | Halidrys siliquosa Extract | Imperata cylindrica Root Extract | Codium tomentosum Extract | Astragalus membranaceus Extract |
|  | Imperata cylindrica Root Extract | Halidrys siliquosa Extract | Halidrys siliquosa Extract | Cacao Seed Extract |
|  | Saccharomyces/Leuconostoc/Apple Fruit/Carrot Root/Radish Root/Cabbage Leaf/Celery Leaf/Cucumber Fruit/Banana Fruit/Onion Bulb/Arctium Lappa Root/Spinach Leaf/ Orange Peel/Tomato Fruit/Phaseolus Radiatus Sprout/Pumpkin Fruit Extract Ferment Filtrate | Saccharomyces/Leuconostoc/Apple Fruit/Carrot Root/Radish Root/Cabbage Leaf/Celery Leaf/Cucumber Fruit/Banana Fruit/Onion Bulb/Arctium Lappa Root/Spinach Leaf/ Orange Peel/Tomato Fruit/Phaseolus Radiatus Sprout/Pumpkin Fruit Extract Ferment Filtrate | Helichrysum italicum Leaf Cell Extract | Chinese matrimony Extract |
|  |  |  | Jania rubens Extract | Chrysanthemum indicum Flower Extract |
|  |  |  | LactococcusFerment Lysate | Dendropanax Morbifera Sap Ferment |
|  |  |  | Lapacho extract | Gleditsia japonica Fruit Extract |
|  |  |  | Opuntia ficus indica Leaf Cell Extract | Lactobacillus/Rice Ferment |
|  |  |  | Polypodium Vulgare Rhizome Extract/ Cetraria Islandica Thallus Extract/ | LactococcusFerment Lysate |
|  |  |  | Portulaca oleracea Extract | Lapacho extract |
|  |  |  |  | Ligustrum japonicum Fruit |
|  |  |  |  | Lonicera japonica (Honeysuckle) Flower Extract |
|  |  |  |  | Lycium Chinense Fruit Extract |
|  |  |  |  | Macadamia integrifolia Seed Oil |
|  |  |  |  | Nelumbo nucifera Seed Extract |
|  |  |  |  | Ophiopogon japonicus Extract |
|  |  |  |  | Panax Ginseng Seed Extract |
|  |  |  |  | Polygonum multiflorum Root Extract |
|  |  |  |  | Polypodium Vulgare Rhizome Extract/ Cetraria Islandica Thallus Extract/ |
|  |  |  |  | Prunus Armeniaca (Apricot) Kernel Extract |
|  |  |  |  | Schisandra chinensis Fruit Extract |
|  |  |  |  | Solomon's seal Root Extract |
|  |  |  |  | Aquilaria agallocha Stem Extract |

**Supplementary Table 3.** Compositions of moisturizing agents used for the preparation of the basic cosmetics, skin softener, lotion, essence, and cream.

| **Cosmetics/type** | **Humenctant** | **Content in Cosmetics (%)** | **Content in ingredient (%)** |
| --- | --- | --- | --- |
| **Skin softener: Solubilized type** | PEG/PPG-17/6 Copolymer | 2.5 | 100 |
|  | Glycerin | 2.0 | 100 |
|  | Water | 1.0 | 89.85 |
|  | Polyphosphorylcholine Glycol Acrylate |  | 5.00 |
|  | Butylene Glycol |  | 5.00 |
|  | Methylparaben |  | 0.15 |
|  | Methyl Gluceth-20 | 0.5 | 100 |
|  | Glycerin | 4.0 | 30.00 |
|  | Butylene Glycol |  | 20.00 |
|  | Water |  | 17.90 |
|  | Betaine |  | 15.00 |
|  | Sodium Hyaluronate |  | 15.00 |
|  | Imperata cylindrica Root Extract |  | 1.50 |
|  | PEG-8 |  | 0.30 |
|  | Carbomer |  | 0.20 |
|  | Methylparaben |  | 0.10 |
|  | Water | 0.5 | 97.60 |
|  | Biosaccharide Gum-1 |  | 1.10 |
|  | Phenoxyethanol |  | 1.2160 |
|  | Methylparaben |  | 0.0480 |
|  | Ethylparaben |  | 0.0120 |
|  | Butylparaben |  | 0.0120 |
|  | Propylparaben |  | 0.0060 |
|  | Isobutylparaben |  | 0.0060 |
| **Lotion: O/W emulsion type** | Glycerin | 2.0 | 100 |
|  | Glycerin | 10 | 30.00 |
|  | Butylene Glycol |  | 20.00 |
|  | Water |  | 17.90 |
|  | Betaine |  | 15.00 |
|  | Sodium Hyaluronate |  | 15.00 |
|  | Imperata cylindrica Root Extract |  | 1.50 |
|  | PEG-8 |  | 0.30 |
|  | Carbomer |  | 0.20 |
|  | Methylparaben |  | 0.10 |
|  | Sodium Hyaluronate | 0.04 | 100 |
| **Essence: Solubilized type** | Glycerin | 1.5 | 100 |
|  | Propandiol | 3.0 | 100 |
|  | PEG-75 | 0.7 | 100 |
|  | Sodium Hyaluronate | 0.04 | 100 |
|  | Hydroxyethyl Urea | 1.0 | 100 |
|  | Lactococcus Ferment Lysate | 0.1 | 100 |
|  | Propanediol | 0.1 | 58.45 |
|  | Water |  | 37.50 |
|  | Dryopteris Filix-Mas Root Extract |  | 3.00 |
|  | Cetraria islandica Extract |  | 0.50 |
|  | Peat Extract |  | 0.50 |
|  | Citric Acid |  | 0.05 |
|  | Butylene Glycol | 0.1 | 49.50 |
|  | Water |  | 48.90 |
|  | Codium tomentosum Extract |  | 1.60 |
|  | Glycerin | 0.1 | 40–60 |
|  | Water |  | 40–60 |
|  | Tabebuia Impetiginosa Bark Extract |  | 1–3 |
|  | Sodium Benzoate |  | 0.2–0.3 |
|  | Potassium Sorbate |  | 0.2–0.3 |
| **Cream: O/W emulsion type** | Theobroma Cacao (Cocoa) Seed Extract | 0.05 | 100 |
|  | Glycerin | 2.00 | 100 |
|  | Soilum Hyaluoronate | 0.04 | 100 |
|  | Propandiol | 3.00 | 100 |
|  | Lactococcus Ferment Lysate | 0.1 | 100 |
|  | Propanediol | 0.1 | 58.45 |
|  | Water |  | 37.50 |
|  | Dryopteris Filix-Mas Root Extract |  | 3.00 |
|  | Cetraria islandica Extract |  | 0.50 |
|  | Peat Extract |  | 0.50 |
|  | Citric Acid |  | 0.05 |
|  | Butylene Glycol | 0.1 | 49.50 |
|  | Water |  | 48.90 |
|  | Codium tomentosum Extract |  | 1.60 |
|  | Glycerin | 0.1 | 40–60 |
|  | Water |  | 40–60 |
|  | Tabebuia Impetiginosa Bark Extract |  | 1–3 |
|  | Sodium Benzoate |  | 0.2–0.3 |
|  | Potassium Sorbate |  | 0.2–0.3 |
